# Contact and communication: ZO‐2 and the Hippo pathway

**DOI:** 10.1111/febs.17417

**Published:** 2025-02-05

**Authors:** Miranda Thomas

**Affiliations:** ^1^ ICGEB Trieste Italy

**Keywords:** cell communication, Hippo, tight junctions, transcriptional regulation

## Abstract

The PDZ domain‐containing protein ZO‐2 is defined as a tight junction (TJ) protein, but is also known to have a role in the maintenance of cellular apicobasal polarity and to function as a signalling molecule in several pathways, including the Hippo pathway. In this issue, Liu OX *et al.* [(2024) *FEBS J*, https://doi.org/10.1111/febs.17304] report how the multiple protein binding sites of ZO2 protein allow it to act as a scaffold to facilitate its signalling functions.

## Graphical abstract

The PDZ domain‐containing protein ZO‐2 is defined as a tight junction protein, but is also known to play a role in the maintenance of cellular apicobasal polarity, and to function as a signalling molecule in several pathways, including the Hippo pathway. Liu *et al*. report how the multiple protein binding sites of ZO‐2 protein enable it to act as a scaffold, thereby facilitating its signalling functions.

Comment on: https://doi.org/10.1111/febs.17304.

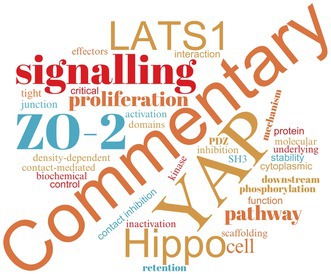

**Abbreviations**
AJ: adherens junction
JAMs: junctional adhesion molecules
NES: nuclear export sequence
PBM: PDZ‐binding motif
TJ: tight junction

How cells detect the presence of other cells, and how they communicate information about cell density, both intra‐ and inter‐cellularly is an extremely intriguing question that has excited many researchers. The confluence of different internal signalling pathways, in particular those regulating planar and apicobasal polarity, with those regulating cell proliferation, forms vital signalling nodes in multicellular tissues.

The Hippo signalling pathway controls organ size and is involved in restraining cell proliferation and promoting apoptosis. It also plays a critical role in stem cell self‐renewal and expansion [1]. Thus, the correct functioning of the Hippo pathway is essential for normal tissue homeostasis, and as many cancers probably originate in tissue‐specific stem cells and are characterised by unrestrained cell division, any perturbations of Hippo function are highly important. The active YAP and TAZ transcriptional coactivators collaborate with the DNA sequence‐specific TEAD transcription factors in the nucleus to promote cell proliferation by activating the expression of a number of proliferation‐related genes. They are negatively regulated through the Hippo pathway, where the MST1 and MST2 kinases phosphorylate, and thereby activate, the LATS kinases, which in turn phosphorylate YAP and TAZ. This phosphorylation promotes YAP and TAZ's binding to 14‐3‐3 proteins, which tethers them in the cytoplasm, preventing their transactivation activity.

Tight junctions (TJs) are essential, together with adherens junctions (AJs), for forming the barrier functions of epithelial and endothelial cells; they occur in a band (zonula occludens, ZO) around the cell, forming regions of contact between cells. They are multi‐protein complexes that occur at the apical part of the lateral membrane, and comprise multiple transmembrane proteins, including occludin, claudins, junctional adhesion molecules (JAMs) and anguilins, that form extracellular contacts with their counterparts in neighbouring cells [2]. In addition to their barrier functions in the epithelium, TJs can also form selectively permeable channels, especially between endothelial cells, to allow specific movement of molecules. TJs play a vital role in the establishment of apicobasal polarity in epithelial cells: components of the apical polarity complexes interact with the ZO proteins and JAM‐A, to mutually control the localisation of the apical Crumbs complex and the basolateral Scribble (Scrib) complex to the apical and lateral regions, respectively.

Most importantly for the purposes of this discussion, several TJ proteins are involved in signalling—connecting input regarding the status of the cell surface, including the TJ itself, to transcriptional or cytoskeletal response mechanisms, including the Hippo pathway. The TJ proteins ZO‐1 and ZO‐2 are PDZ domain‐containing proteins that play essential roles in the proper assembly and maintenance of the TJ [3]. They are also signalling molecules that interact with a number of different signalling pathways, including the Hippo pathway [2, 4] and ZO‐1 has also been shown to regulate certain Hippo‐independent activities of YAP [5]. However, ZO‐2 has been dubbed a ‘master regulator’ of a number of cellular functions, including gene expression [6]. In addition, in cells, such as fibroblasts, that lack TJs, ZO‐2 proteins locate to the AJs [7], underlining the importance to cellular homeostasis of ZO‐2's non‐TJ‐related functions.

The interaction of ZO‐2 with proteins of the Hippo pathway has long been known, but the precise effects of this interaction have been debated. YAP has a C‐terminal PDZ‐binding motif (PBM) that specifically binds the first PDZ domain of ZO‐2, which was proposed to thereby facilitate the nuclear localisation of YAP, presumably via the ZO‐2 nuclear localisation signal [8, 9]. However, it was also shown that silencing ZO‐2 results in increased YAP nuclear accumulation and transcriptional activity in MDCK cells [10]. It is possible that this apparent inconsistency is related to differences in the systems used to examine this question, as canine ZO‐2 contains four nuclear export sequences (NES) that human ZO‐2 lacks, and therefore, the nuclear export of ZO‐2/YAP may occur through different mechanisms depending on the cell type/species.

The paper by Liu *et al*. in this issue of *The FEBS Journal* [11] adds an additional dimension to this debate—bringing the scaffolding function of ZO2 into the equation. In agreement with previous publications, they show that the YAP PBM binds to the PDZ1 domain of ZO‐2. They also show that the stability of LATS1, which phosphorylates YAP, depends on ZO‐2's inhibition of LATS1 polyubiquitination and subsequent proteomic degradation. LATS1 itself can bind to the SH3 domain of ZO‐2, which is distant from the PDZ1 domain, thus allowing both LATS1 and YAP to bind simultaneously to ZO‐2, forming a trimer that brings active LATS1 into close proximity with YAP, which it then can phosphorylate.

Thus, in nonconfluent cells, a pool of free ZO‐2 may facilitate the nuclear entry of YAP, which can then coactivate transcription of proliferation‐related genes. When the cell reaches confluence, ZO‐2 dissociates from the TJ, binding and stabilising activated LATS1, as well as binding YAP. Bringing YAP and LATS1 together results in phosphorylated YAP, which binds 14‐3‐3 and is tethered in the cytoplasm and prevented from co‐activating transcription, thereby preventing cell proliferation.

This is potentially at least part of the mechanism through which signalling from the TJs influences cell proliferation and controls tissue homeostasis.

Although this paper reports a clear and convincing demonstration of the importance of the scaffolding functions of PDZ domain‐containing proteins, a number of questions remain to be investigated. These experiments were performed in a monolayer culture of MDCK cells, which are known to have particularly discrete cell junctions and strong ZO protein localisation to those junctions. It would be interesting to know whether similar results are seen in other simple epithelia that have more diffuse cell junctions, and how this signalling is modified in different layers of a differentiated epithelium, or between the mixture of different cell types found in organs.

Another clear message from this paper is the importance of proper cell junctional formation for the correct localisation of signalling molecules and thus for the transmission of signals. The paper demonstrates that junctions that form too quickly lack the correct structure and are thus defective in their ability to send the correct signals regarding cell confluence. This is an important point, since cancer cells grow more quickly than normal cells, they are likely to have inadequately matured junctions and thus be incapable of correctly signalling that the cells have reached confluence. This would be a self‐reinforcing cycle since it would result in the continued expression of proliferation‐related genes and thus in further increased cell growth. It is probable that such a reinforcement cycle may play a role in the development of certain cancers.

## Conflict of interest

The author declares no conflict of interest.
